# Grass is not always greener: rodenticide exposure of a threatened species near marijuana growing operations

**DOI:** 10.1186/s13104-018-3206-z

**Published:** 2018-02-02

**Authors:** Alan B. Franklin, Peter C. Carlson, Angela Rex, Jeremy T. Rockweit, David Garza, Emily Culhane, Steven F. Volker, Robert J. Dusek, Valerie I. Shearn-Bochsler, Mourad W. Gabriel, Katherine E. Horak

**Affiliations:** 1 USDA-APHIS-WS National Wildlife Research Center, 4101 Laporte Ave, Fort Collins, CO 80521 USA; 2 Colorado Cooperative Fish and Wildlife Research Unit, Colorado State University, 1484 Campus Delivery, Fort Collins, CO 80523 USA; 3 U. S. Geological Survey, National Wildlife Health Center, 6006 Schroeder Road, Madison, WI 53711 USA; 4 Integral Ecology Research Center, 239 Railroad Avenue, Blue Lake, CA 95525 USA

**Keywords:** Brodifacoum, Rodenticide, Spotted owl, Marijuana, *Cannabis*, Secondary poisoning, California, Toxicant

## Abstract

**Objective:**

Marijuana (*Cannabis* spp.) growing operations (MGO) in California have increased substantially since the mid-1990s. One environmental side-effect of MGOs is the extensive use of anticoagulant rodenticides (AR) to prevent damage to marijuana plants caused by wild rodents. In association with a long-term demographic study, we report on an observation of brodifacoum AR exposure in a threatened species, the northern spotted owl (*Strix occidentalis caurina*), found freshly dead within 669–1347 m of at least seven active MGOs.

**Results:**

Liver and blood samples from the dead northern spotted owl were tested for 12 rodenticides. Brodifacoum was the only rodenticide detected in the liver (33.3–36.3 ng/g) and blood (0.48–0.54 ng/ml). Based on necropsy results, it was unclear what role brodifacoum had in the death of this bird. However, fatal AR poisoning has been previously reported in owls with relatively low levels of brodifacoum residues in the liver. One likely mechanism of AR transmission from MGOs to northern spotted owls in California is through ingestion of AR contaminated prey that frequent MGOs. The proliferation of MGOs with their use of ARs in forested landscapes used by northern spotted owls may pose an additional stressor for this threatened species.

**Electronic supplementary material:**

The online version of this article (10.1186/s13104-018-3206-z) contains supplementary material, which is available to authorized users.

## Introduction

The number and extent of marijuana (*Cannabis* spp.) growing operations (MGO) in California have increased substantially since the mid-1990s, with a mix of illegal clandestine operations and those growing for medical or, recently, recreational use [1–3]. California is the largest producer of marijuana in the U.S., with Humboldt, Trinity and Mendocino Counties the epicenter for production [4]. There are ~ 15,000 documented MGOs in Humboldt County [5] and an estimated 4428 of these were visible outdoor MGOs on private lands (either as greenhouses, crop fields, or both) in 53.6% of the watersheds [3]. One environmental side-effect of marijuana production in California is the extensive use of anticoagulant rodenticides (AR) to prevent damage to plants caused by wild rodents [6, 7]. Oftentimes, substantial amounts of AR (up to ~ 25 kg) are found at illegal MGOs on public lands [6–9]. Although a large number of MGOs on private lands are quasi-legal in California, the distinction between illegal and legal operations for enforcement purposes is difficult. Because marijuana is still federally illegal, no pesticides are registered for its use as an agricultural crop [10]. For these reasons, regulatory compliance on quasi-legal MGOs is uncertain and assumed to be low. Enforcement of regulations by government agencies is minimal because of the sheer number of unpermitted MGOs and remoteness of these operations [3, 10, 11]. For example, only 15% of MGOs in Humboldt County have applied for permits with only 0.6% approved, suggesting that over 85% of MGOs in the county have not applied to be under regulatory compliance [5]. Thus, AR use on MGOs in California is probably ubiquitous, regardless of legality, because of the perceived threat of wild rodent damage to marijuana crops on MGOs and the lack of permitting and enforcement [12, 13].

Secondary poisoning of non-target wildlife (species unintentionally exposed to AR) from ARs has become a re-emerging threat in California, especially around outdoor MGOs on or near public lands, which are considered a primary source of AR in wild environments [6, 7, 9, 14]. For example, dead wildlife from AR poisoning were found at 21.9% of 41 MGOs investigated in Humboldt, Trinity, and Siskiyou counties; these included bears, foxes, fisher (*Pekania pennant*), squirrels, deer, and passerine birds [15]. In addition, liver residues in wild rodents at MGOs also tested positive for ARs [15]. Although raptors found dead with signs of AR poisoning were not found at MGOs, they prey on rodents affected by AR at MGOs and possibly die elsewhere. ARs in prey presents a risk to owls that subsequently bioaccumulate ARs in tissues, especially the liver [16], taking up to 15 days to produce lethal concentrations. Thus, owls are at high-risk for secondary AR poisoning because of their specialization on rodent prey [16]. For example, 62–90% of carcasses from three owl species in western Canada had detectable residues from ≥ 1 AR, even death from AR poisoning was determined in only 2–12% of cases [17].

Of particular concern is the threat of secondary AR poisoning in northern spotted owls (*Strix occidentalis caurina*), a threatened species under the Endangered Species Act [18] currently experiencing 2.3–3.0% annual population declines in California due to a number of stressors [19]. MGOs occur within areas used by northern spotted owls, which have the potential to be exposed to ARs through their primary prey in northern California, dusky-footed woodrats (*Neotoma fuscipes*). Dusky-footed woodrats are also perceived by growers as a threat to marijuana plants because they forage on young plants in the spring and use the plants to build nests [12, 13]. Recently, 70% of northern spotted owls found dead had evidence of being exposed to ARs, with the hypothesis that increased MGOs on the landscape were the primary source [20]. However, AR use on MGOs has not been explicitly linked with northern spotted owl exposures.

**Fig. 1:**
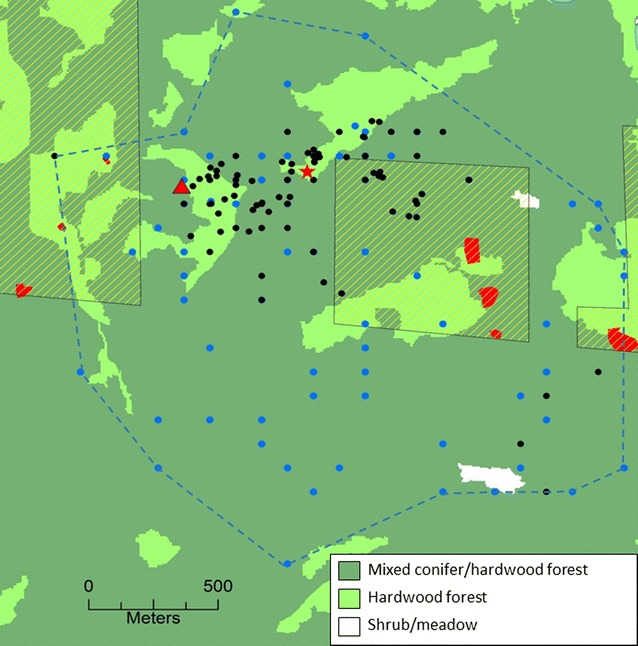
Location of dead female northern spotted owl (red star) on an established territory (blue dashed line) in Humboldt County, California in proximity to known marijuana growing operations (red polygons). An illegal clandestine MGO eliminated in 2015 is shown as a red triangle. Yellow-hatched areas are private inholdings in a national forest. Black dots are roost and nest sites used by northern spotted owls and blue dots are nocturnal detections of spotted owls from surveys conducted on this territory from 1985 to 2017. Aerial views of MGOs can be seen in [3, 41]

In association with a long-term demographic study, we report on brodifacoum exposure in a northern spotted owl found freshly dead in the vicinity of at least 7 active MGOs on private inholdings within a National Forest (Fig. 1) during a routine survey to detect owls. Finding a freshly dead northern spotted owl in the woods is a very rare event; this is the first time we have encountered a recently deceased adult during 9216 foot surveys on 95 spotted owl territories over a 33-year study. Thus, this observation is important in establishing a potential linkage that warrants further research to determine the magnitude of this threat to northern spotted owl populations.

## Main text

### Methods

A female northern spotted owl was found dead 5 April 2017 in a territory that had been monitored since 1985 [21] south of Willow Creek, Humboldt County, California. The female was marked with a USGS numbered band and a unique colored band in 2008 and had occupied the territory since 2016. The female was estimated to be dead ≤ 24 h because (1) the carcass was fresh with the eyes not sunken, (2) there were no fly larvae on the carcass, and (3) the male owl attempted to deliver a mouse to the carcass for ~ 5 min.

The carcass was collected and shipped chilled to the USGS National Wildlife Health Center (NWHC), Madison, WI for necropsy. Liver and blood samples were taken and sent for AR residue analysis at the USDA-APHIS National Wildlife Research Center, Fort Collins, CO.

The liver and blood samples were subsequently tested for 11 anticoagulant rodenticides (coumafuryl, coumatetralyl, pindone, warfarin, coumachlor, diphacinone, chlorophacinone, bromadiolone, difenacoum, brodifacoum, difethialone) and 1 neurotoxicant rodenticide metabolite (desmethyl bromethalin) (see Additional file 1 for exact methods).

To identify potential sources of AR, we first delineated the potential foraging area for northern spotted owls on this particular territory using night and day locations of northern spotted owls detected during surveys conducted from 1985 to 2017 during our demography study [22]. Following [3], we scanned high-resolution satellite imagery from May 2016 in Google Earth^®^ (www.google.com/earth/) around the polygon formed by the owl locations to search for visible AR sources, such as residences and MGOs, within this particular owl territory. We also included locations of illegal MGOs found by law enforcement agencies (M. Gabriel, unpublished data). All MGOs were known to local law enforcement authorities. We were unable to determine whether the MGOs identified were using ARs. As one study noted [23], most landowners with MGOs are unwilling to allow visitors to their operation and it is usually dangerous for researchers to approach them. All locational data was entered into a geographic information system (ArcGIS 10^®^) to develop maps (e.g., Fig. 1) and measure distances from the owl mortality site and potential sources of AR poisoning. Because of legal restrictions, specific locational information for either the spotted owl or MGOs on private lands cannot be provided.

### Results and discussion

The necropsy found that the female was reproductively active with developing follicles in the ovary, was emaciated, and heavily parasitized with large numbers of *Leucocytozoon* spp. protozoa in red blood cells and *Elmeria* spp., coccidia and *Capillariid* spp. in the intestine. The female weighed 490 g when found, which was 73.9% of the mean weight for female northern spotted owls [24]; no baseline weight had been taken for this individual when first captured in 2008. No other abnormalities, including trauma, were detected. The owl tested negative for avian influenza viruses, West Nile virus, and exposure to lead. Brain cholinesterase levels were not depressed, suggesting no acute exposure to cholinesterase-inhibiting toxicants, such as organophosphate or carbamate pesticides. Proximate cause of death was diagnosed as emaciation and parasitism.

Brodifacoum was the only rodenticide detected in the liver (33.3–36.3 ng/g) or the blood (0.48–0.54 ng/ml) (Table 1). In this case, exposure to brodifacoum was not the primary cause of death of the northern spotted owl examined here, as there was no sign of internal hemorrhage indicative of AR poisoning. However, the levels of brodifacoum residues found in the owl’s liver have been associated with lethal AR poisoning in other owl species, such as great horned owls (*Bubo virginianus*) with liver residues as low as 10 ng/g [25]. Two northern spotted owls submitted to the NWHC in the 1990s had brodifacoum liver residues of 50.0 and 100 ng/g and signs of hemorrhaging (NWHC Case Numbers 10128 and 13799). Although not directly linked as the cause of mortality, exposure to brodifacoum in the owl we found may have resulted in a sub-lethal exposure. While sub-lethal effects of brodifacoum on non-target wildlife are poorly understood, they are hypothesized to include anemic lethargy that impairs hunting ability leading to loss of body mass, and increased susceptibility to disease [26, 27]. For example, sub-lethal exposures to brodifacoum slowed growth in Japanese quail [28] and was associated with an outbreak of notoedric mange in bobcats (*Lynx rufus*), which led to a 64% reduction in survival [29]. Combined with the northern spotted owl’s reproductive status and heavy parasitism, brodifacoum may have been an additional contributor to the owl’s death.

**Table 1:** Rodenticide analysis of liver and blood from deceased northern spotted owl

Rodenticide	Observed concentration		Detection limit^a^		Quantitation limit^b^	
	Liver^c^ (ng/g)	Blood^c^ (ng/ml)	Liver (ng/g)	Blood (ng/g)	Liver (ng/g)	Blood (ng/g)
Brodifacoum	33.3, 36.3, 35.7	0.54, 0.48, ND	5.80	0.45	19.30	1.48
Bromadiolone^d^	ND	ND	0.59, 0.78	0.09, 0.13	1.96, 2.59	0.28, 0.42
Bromethalin^e^	ND	ND	5.10	0.41	17.00	1.37
Chlorophacinone	ND	ND	13.00	0.28	42.50	0.95
Coumachlor	ND	ND	0.33	0.03	1.09	0.09
Coumatetralyl	ND	ND	8.80	0.60	29.20	1.99
Coumafuryl	ND	ND	2.40	0.23	8.11	0.76
Difenacoum	ND	ND	27.00	3.30	89.80	11.00
Difethialone	ND	ND	4.50	0.25	15.1	0.84
Diphacinone	ND	ND	8.50	1.10	28.40	3.53
Pindone^f^	ND	ND	75.00	10.00	NE	NE
Warfarin	ND	ND	1.80	0.20	5.90	0.68

^a^Detection limit (DL) is the lowest concentration of analyte in a sample that can be detected but not necessarily quantified as an exact concentration

^b^Quantitation limit (QL) is the lowest concentration of analyte that can be quantified with suitable precision and accuracy

^c^Results are either from triplicate replications or not detected (ND)

^d^Values under DL and QL are for bromadiolone A and B, respectively

^e^Tested for the metabolite desmethyl bromethalin

^f^Estimated based on previous multi-rodenticide analyses; NE = no estimate

Brodifacoum is commonly used in household, industrial and agricultural settings and is often found at illegal clandestine MGOs [5–8] to prevent rodents from damaging the stalks of marijuana plants where it is applied at plant bases and around MGO perimeters [6]. The dead northern spotted owl was found within 669–1347 m of at least 7 active MGOs on private inholdings within a National Forest (Fig. 1). In 2015, an illegal clandestine MGO with ~ 23 kg of brodifacoum-laced bait was discovered 450 m from the recovery location of this dead owl (M. Gabriel, unpublished data, Fig. 1), indicating that other undetected MGOs may have been nearby. Given the documented use of brodifacoum on MGOs, it is highly likely that the source of the brodifacoum residues found in the dead owl were from one or more of the MGOs within its territory (Fig. 1); we were unable to identify any other potential sources.

One probable mechanism of AR transmission from MGOs to northern spotted owls in this region of California is through ingestion of dusky-footed woodrats, which are a dominant prey of spotted owls in this area [30]. Dusky-footed woodrats are abundant in early-seral stages, such as openings created by fire, timber harvests, or (presumably) MGOs, and have specialized gut microbiomes that allow them to digest toxic secondary plant compounds and fibrous plant material [31], typical of marijuana plants. In addition, dusky-footed woodrats incorporate plants with high monoterpene content [32, 33], such as California bay (*Umbellularia californica*), into their nests, with evidence that these plants act as larvicides and repellants against fleas [34, 35]. *Cannabis* contains similar monoterpenes and can also act as a larvicide against mosquito and other insect larvae [36–38]; anecdotal observations indicate that woodrats incorporate marijuana stalks into their nests [13]. Both California bay and marijuana plants are aromatic [34, 39] and woodrats may be able to detect these on the landscape through olfactory cues. Northern spotted owls in California also tend to forage near edges of openings when woodrats predominate in their diet [40]. In forested landscapes in Humboldt County, California, MGOs have generated increased edge with forest areas and increased patch shape complexity [41], landscape elements that also contribute to high-quality habitat for northern spotted owls [30].

**Fig. 2:**
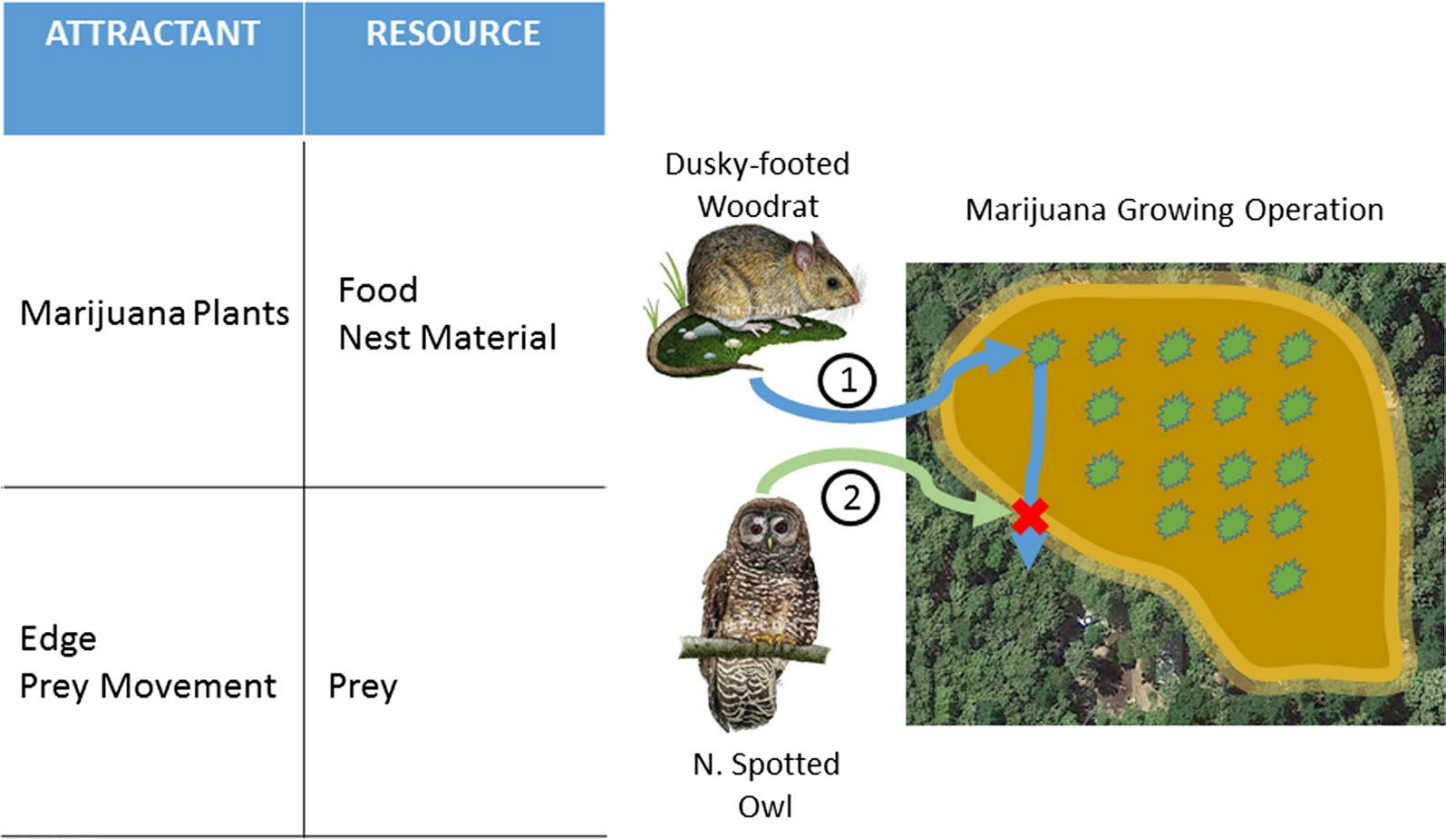
Hypothesized mechanism for exposure of northern spotted owls to anticoagulant rodenticides (AR) used on marijuana growing operations (MGOs) in northern California. Dusky-footed woodrats are attracted to marijuana plants on MGOs for food and larvicidal nest material (pathway 1). Woodrats are exposed to ARs and return to forested habitat where they are encountered (red X) by northern spotted owls foraging (pathway 2) along forest edge (yellow stippled area)

Based on this, we propose a mechanistic hypothesis on the linkage between MGOs and secondary AR poisoning of northern spotted owls (Fig. 2), where woodrats are attracted to MGOs by the presence of marijuana plants for food and larvicidal nest material and encounter ARs in rodent baits while foraging on MGOs. Northern spotted owls are also attracted to MGOs because of the edge habitat created by MGOs and increased prey movement across those edges. This edge becomes the area where owls then prey on AR-contaminated woodrats.

### Conclusions

The observation of a northern spotted owl with AR residues in proximity to numerous MGOs further suggests the potential linkage between MGOs and AR exposure for this threatened species as another additive stressor. Thus, outdoor MGOs in forested landscapes may provide resources for prey and foraging opportunities for northern spotted owls but with potentially lethal consequences for both.

### Limitations

We were unable to definitely identify MGOs as the source for the brodifacoum found in the dead northern spotted owl. However, we were not able to identify any other potential sources of AR within the territory used by this particular owl. Thus, our case study provides evidence to support the hypothesis that MGOs may constitute an additional threat to northern spotted owl populations in northwestern California, a hypothesis that should be examined with further research.

## Additional file

**Additional file 1.** Analytical methods for rodenticide detection and quantitation.

## Abbreviations

AR: anticoagulant rodenticides
MGO: marijuana (*Cannabis* sp.) growing operation
NWHC: USGS National Wildlife Health Center
USGS: U. S. Geological Survey
