# Supplementary material for: Grass is not always greener: rodenticide exposure of a threatened species near marijuana growing operations
Source: BMC Res Notes. 2018 Feb 2;11:94. doi: 10.1186/s13104-018-3206-z (PMC5796583; doi:10.1186/s13104-018-3206-z)
Supplement: Supplementary file 1 — Additional file 1. Analytical methods for rodenticide detection and quantitation. [file 13104_2018_3206_MOESM1_ESM.docx]

**Additional file 1.** Analytical methods for rodenticide detection and quantitation

**Materials**

Coumafuryl, warfarin, coumachlor, bromadiolone, difenacoum, and flocoumafen were obtained from Fluka Chemicals, St. Louis, MO. Diphacinone was purchased from Hacco Inc., Randolph, WI. Coumatetralyl and brodifacoum were purchased from Sigma-Aldrich, St. Louis, MO. Chlorophacinone and difethialone were obtained from LiphaTech, Inc., Milwaukee, WI. Desmethyl bromethalin was purchased from Toronto Research Chemicals, Toronto, Ontario, Canada. D_5_-warfarin, D_4_-diphacinone, D_4_-chlorophacinone, and D_5_-bromadiolone were purchased from CDN Isotopes, Inc., Pointe-Claire, Quebec, Canada. A chlorine substituted analog of brodifacoum (hereafter referred to as chlordifacoum) was obtained from Richman Chemical Inc., Lower Gwynedd, PA. 1.5-mL polypropylene microcentrifuge tubes were purchased from Molecular BioProducts, Inc., San Diego, CA. 2-mL dispersive solid phase extraction (dSPE) tubes were purchased from Agilent Technologies, Santa Clara, CA. HPLC grade acetonitrile (ACN) and USP grade sodium chloride (NaCl) were obtained from Thermo Fisher Scientific, Hampton, NH. Control quail were purchased from Rodentpro.com, Evansville, IN, and control whole chicken blood (with 0.5% sodium citrate) from Pel-Freez Biologicals, Rogers, AR.

**Sample Preparation**

Liver: Liver was homogenized using a SPEX CertiPrep 6850 Freezer/Mill® Cyrogenic Grinder (Metuchen, NJ, USA) and 70-80 mg samples were weighed into microcentrifuge tubes. DI water (0.050 mL) was added and the sample vortex mixed to form a uniform suspension. Surrogate analytes were added (0.020 mL ACN containing 7 µg/mL flocoumafen and 17 µg/mL each of D_5_-warfarin, D_4_-diphacinone, D_4_-chlorophacinone, D_5_-bromadiolone, and chlordifacoum). ACN was added (1.18 mL) and the sample vortex mixed 20-30 s. Excess NaCl was added (~120 mg) and the sample vortex mixed again, resulting in phase separated water and ACN. The extract was centrifuged at 12,000 RCF for 1 minute and 0.90 mL of the upper phase transferred to a dSPE tube containing MgSO_4_ (150 mg), C_18_ sorbent (25 mg), and primary-secondary amine (PSA) sorbent (25 mg) [1]. The mixture was vortex mixed 4-5 s, and then briefly centrifuged at 12,000 RCF to clarify the extract. A portion of the supernatant (0.40 mL) was transferred to a microcentrifuge tube and reduced to dryness with a gentle flow of N_2_ gas in a 45°C water bath. The analytes were reconstituted with 0.100 mL ACN (mobile phase B) followed by 0.400 mL pH 9.5 20-mM ammonium acetate (mobile phase A), and analyzed by LC-MS/MS. Method accuracy and precision results for control quail liver fortified with three rodenticide concentration levels (n=3 at each level) are presented in Table 2. Detection and quantitation limits (Table 4) were estimated from 3X and 10X signal-to-noise in quail liver, respectively.

Whole blood: The whole blood sample (0.5 mL) had been preserved in 3-4 mL of 70% isopropanol. The isopropanol and excess water were removed with a gentle flow of N_2_ gas in a 45°C water bath by twice reducing the volume to ~0.4 mL and reconstituting to 0.5 mL with DI water. The surrogate analyte mixture (as described for liver sample preparation) and 1.18 mL ACN were added and the sample vortex mixed twice for 30-45 s. Excess NaCl (~250 mg) was added to phase separate water and ACN, and the sample centrifuged at 12,000 RCF for 1 minute. A portion of the supernatant (0.40 mL) was reduced to dryness, reconstituted in mobile phase, and analyzed by LC-MS/MS. Method accuracy and precision results for control whole chicken blood fortified with three rodenticide concentration levels (n=3 at each level) are presented in Table 3. Detection and quantitation limits (Table 4) were estimated from 3X and 10X signal-to-noise in control chicken blood, respectively.

**LC-MS/MS Analysis**

Each sample (7.5 µL) was injected into an Agilent LC-MS/MS consisting of a 1290 Infinity II UPLC coupled to a G6470A triple-quadrupole mass spectrometer with Jet Stream® electrospray ionization (AJS ESI) source. The analytes were chromatographed at 0.800 mL/min on an Xbridge C18 column (2.5-µm particle size, 2.1 x 50 mm, Waters Corp., Milford, MA, USA) heated to 60°C. Mobile phase A was pH 9.5 20-mM ammonium acetate and mobile phase B was ACN. The gradient program was held at 10% mobile phase B for 0.25 min, increased linearly to 64% over 4.5 min, held at 100% for 1 min to clean the column, and then re-equilibrated at 10% B for 1.25 min. The source drying gas (N_2_) was 300°C (5 L/min), nebulizer pressure 45 psi, sheath gas 7 L/min at 250°C, nozzle 0 V, and capillary voltage -4000 V. Two mass transitions were monitored for each of the 12 analytes and one transition for each of the six surrogate analytes (Table 1). The analyte to surrogate peak area response ratio (6-level standard curve for each analyte ranging from approximately 1 to 700 ng/mL) was used to quantify samples. Each calibration curve was fit to either a quadratic or second order logarithmic function and achieved correlation coefficients (R^2^) ranging from 0.9958 to 0.9999. Attempts to quantify bromethalin were unsuccessful. Instead, the method quantifies the metabolite desmethyl bromethalin [2]. Pindone was not available at the time of testing, however the MS/MS transitions, retention time, and an approximate detection limit was known from previous unpublished studies.

| Table 1. Agilent G6470B MS/MS conditions for 12 rodenticides (and six surrogates) | | | | | |
| --- | --- | --- | --- | --- | --- |
|  |  |  |  |  |  |
|  | Precursor | Product | Fragmentor | Collision | Retention |
| Analyte | Ion (m/z) | Ion (m/z) ^a^ | (V) | Energy (V) | Time (min) |
| Coumafuryl | 297.0 | **161.0** | 80 | 10 | 0.7 |
|  |  | 240.1 |  | 13 |  |
| D_5_-Warfarin * | 312.0 | **160.9** | 92 | 15 | 1.3 |
| Warfarin | 307.1 | **250.1** | 100 | 11 | 1.4 |
|  |  | 161.0 |  | 18 |  |
| Pindone | 229.1 | **116.0** | 110 | 33 | 1.4 |
|  |  | 144.1 |  | 24 |  |
| Coumatetralyl | 291.0 | **141.1** | 100 | 20 | 1.5 |
|  |  | 247.1 |  | 26 |  |
| Coumachlor | 341.0 | **284.0** | 95 | 20 | 1.9 |
|  |  | 161.0 |  | 16 |  |
| D_4_-Diphacinone * | 343.1 | **167.1** | 120 | 23 | 2.5 |
| Diphacinone | 339.1 | **167.1** | 100 | 23 | 2.5 |
|  |  | 145.0 |  | 18 |  |
| D_4_-Chlorophacinone * | 377.0 | **201.1** | 110 | 26 | 2.9 |
| Chlorophacinone | 373.1 | **201.1** | 110 | 23 | 2.9 |
|  |  | 145.1 |  | 20 |  |
| D_5_-Bromadiolone * | 529.9 | **254.9** | 212 | 40 | 3.3, 3.5 ^b^ |
| Bromadiolone | 524.9 | **250.0** | 168 | 37 | 3.3, 3.5 ^b^ |
|  |  | 181.0 |  | 27 |  |
| Difenacoum | 443.0 | **293.1** | 158 | 35 | 3.4 |
|  |  | 143.0 |  | 55 |  |
| Chlordifacoum * | 477.1 | **135.1** | 61 | 37 | 3.7 |
| Brodifacoum | 522.9 | **80.9** | 165 | 50 | 3.8 |
|  |  | 135.0 |  | 44 |  |
| Flocoumafen * | 541.0 | **382.1** | 157 | 23 | 3.8 |
| Difethialone | 536.9 | **151.0** | 180 | 37 | 4.0 |
|  |  | 371.1 |  | 42 |  |
| Desmethyl Bromethalin ^c^ | 561.6 | **277.8** | 140 | 35 | 4.8 |
|  |  | 452.9 |  | 30 |  |

* Surrogate analyte ^a^ **Bold** = Quantifier transition ^b^ Stereoisomers

| Table 2. Rodenticide recoveries – Fortified control quail liver | | | | | | | |
| --- | --- | --- | --- | --- | --- | --- | --- |
|  | Surrogate Analyte ^a^ | n | Fortification Levels (ng/g) | Range (%) | Mean (%) | Std Dev. (%) | CV (%) |
| Coumafuryl | 1 | 9 | 35 - 4200 | 71.8 - 85.1 | 79.6 | 4.1 | 5.2 |
| Warfarin | 1 | 9 | 34 - 4200 | 80.7 - 100 | 94.8 | 6.8 | 7.2 |
| Coumatetralyl | 1 | 9 | 38 - 4600 | 64.7 - 95.6 | 86.1 | 9.8 | 11 |
| Coumachlor | 1 | 9 | 35 - 4300 | 89.2 - 114 | 103 | 10 | 9.7 |
| Diphacinone | 2 | 9 | 35 - 4200 | 80.9 - 99.8 | 95.3 | 5.8 | 6.1 |
| Chlorophacinone | 3 | 9 | 35 - 4300 | 74.9 - 98.1 | 90.8 | 8.2 | 9.0 |
| Bromadiolone | 4 | 9 | 36 - 4400 | 92.8 - 107 | 101 | 4.7 | 4.6 |
| Difenacoum | 5 | 9 | 35 - 4200 | 86.5 - 110 | 95.2 | 6.6 | 6.9 |
| Brodifacoum | 5 | 9 | 34 - 4200 | 77.9 - 90.1 | 85.0 | 4.4 | 5.2 |
| Difethialone | 6 | 9 | 53 - 6500 | 82.1 - 89.5 | 86.7 | 3.1 | 3.6 |
| Desmethyl bromethalin | 6 | 9 | 71 - 8600 | 108 - 129 | 122 | 7.5 | 6.2 |

^a^ D_5_-warfarin (1), D_4_-diphacinone (2), D_4_-chlorophacinone (3), D_5_-bromadiolone (4), chlordifacoum (5), flocoumafen (6).

| Table 3. Analytical recoveries – Fortified control whole chicken blood | | | | | | | |
| --- | --- | --- | --- | --- | --- | --- | --- |
|  | Surrogate Analyte ^a^ | n | Fortification Range (ng/mL) | Range (%) | Mean (%) | Std Dev. (%) | CV (%) |
| Coumafuryl | 1 | 9 | 5.5 - 220 | 101 – 113 | 108 | 4.2 | 3.9 |
| Warfarin | 1 | 9 | 5.4 - 220 | 90.8 – 105 | 100 | 5.5 | 5.5 |
| Coumatetralyl | 1 | 9 | 5.9 - 240 | 81.8 – 97.1 | 90.8 | 6.1 | 6.7 |
| Coumachlor | 1 | 9 | 5.5 - 220 | 99.6 – 107 | 103 | 2.5 | 2.4 |
| Diphacinone | 2 | 9 | 5.4 - 220 | 94.3 – 102 | 96.9 | 2.6 | 2.7 |
| Chlorophacinone | 3 | 9 | 5.5 - 220 | 79.3 – 97.3 | 90.3 | 6.2 | 6.9 |
| Bromadiolone | 4 | 9 | 5.6 - 230 | 93.3 – 106 | 99.8 | 4.3 | 4.3 |
| Difenacoum | 5 | 9 | 5.5 - 220 | 82.0 – 92.0 | 86.8 | 4.0 | 4.6 |
| Brodifacoum | 5 | 9 | 5.4 - 220 | 79.3 – 87.4 | 84.1 | 2.5 | 3.0 |
| Difethialone | 6 | 9 | 8.4 - 340 | 68.0 – 84.7 | 79.5 | 5.2 | 6.5 |
| Desmethyl bromethalin | 6 | 9 | 11 - 450 | 86.7 – 98.4 | 92.3 | 3.8 | 4.1 |

^a^ D_5_-warfarin (1), D_4_-diphacinone (2), D_4_-chlorophacinone (3), D_5_-bromadiolone (4), chlordifacoum (5), flocoumafen (6).

| Table 4. Detection limit (DL) and quantitation limit (QL) | | | | | |
| --- | --- | --- | --- | --- | --- |
|  | Liver (ng/g) | |  | Blood (ng/mL) | |
|  | DL | QL |  | DL | QL |
| Coumafuryl | 2.4 | 8.11 |  | 0.23 | 0.756 |
| Warfarin | 1.8 | 5.90 |  | 0.20 | 0.675 |
| Pindone ^a^ | 75 | N/A |  | 10 | N/A |
| Coumatetralyl | 8.8 | 29.2 |  | 0.60 | 1.99 |
| Coumachlor | 0.33 | 1.09 |  | 0.026 | 0.0856 |
| Diphacinone | 8.5 | 28.4 |  | 1.1 | 3.53 |
| Chlorophacinone | 13 | 42.5 |  | 0.28 | 0.946 |
| Bromadiolone | 0.69 | 2.28 |  | 0.11 | 0.348 |
| Difenacoum | 27 | 89.8 |  | 3.3 | 11.0 |
| Brodifacoum | 5.8 | 19.3 |  | 0.45 | 1.48 |
| Difethialone | 4.5 | 15.1 |  | 0.25 | 0.844 |
| Desmethyl bromethalin | 5.1 | 17.0 |  | 0.41 | 1.37 |

^a^ Pindone was not available at time of testing. Detection limits are estimates from previous unpublished studies.

**References**

1. Lehotay SJ, de Kok A, Hiemstra M, van Bodegraven P: Validation of a fast and easy method for the determination of residues from 229 pesticides in fruits and vegetables using gas and liquid chromatography and mass spectrometric detection. J AOAC Int 2005; 88:595-614.

2. Bautista AC, Woods LW, Filigenzi MS, Puschner B: Bromethalin poisoning in a raccoon (Procyon lotor): diagnostic considerations and relevance to nontarget wildlife. J Vet Diagn Invest 2014; 26:154-157.
